# Dynamics of mass transport during nanohole drilling by local droplet etching

**DOI:** 10.1186/s11671-015-0779-5

**Published:** 2015-02-13

**Authors:** Christian Heyn, Thorben Bartsch, Stefano Sanguinetti, David Jesson, Wolfgang Hansen

**Affiliations:** Institut für Angewandte Physik, Universität Hamburg, Jungiusstr. 11, Hamburg, 20355 Germany; L-NESS and Dipartimento di Scienza dei Materiali, Universitá di Milano Bicocca, Milano, Via Cozzi 5320125 Italy; School of Physics and Astronomy, Cardiff University, Cardiff, CF24 3AA United Kingdom

**Keywords:** Droplet epitaxy, Droplet etching, Semiconductor nanostructures, Nanoholes, Self-assembly, Mass transport, Growth modelling

## Abstract

Local droplet etching (LDE) utilizes metal droplets during molecular beam epitaxy for the self-assembled drilling of nanoholes into III/V semiconductor surfaces. An essential process during LDE is the removal of the deposited droplet material from its initial position during post-growth annealing. This paper studies the droplet material removal experimentally and discusses the results in terms of a simple model. The first set of experiments demonstrates that the droplet material is removed by detachment of atoms and spreading over the substrate surface. Further experiments establish that droplet etching requires a small arsenic background pressure to inhibit re-attachment of the detached atoms. Surfaces processed under completely minimized As pressure show no hole formation but instead a conservation of the initial droplets. Under consideration of these results, a simple kinetic scaling model of the etching process is proposed that quantitatively reproduces experimental data on the hole depth as a function of the process temperature and deposited amount of droplet material. Furthermore, the depth dependence of the hole side-facet angle is analyzed.

## Background

Nanostructuring fundamentally modifies the optoelectronic properties of semiconductor crystals establishing low-dimensional confinements for embedded charge carriers. In particular, self-assembled semiconductor nanostructures are of appreciable interest, since they allow research on well-defined quantum structures without the need for sophisticated lithography. In the field of epitaxial nanostructuring, mainly two self-assembly techniques are utilized: the strain-driven Stranski-Krastanov formation involving, for example InAs [1-3] or Ge [4] nanostrutures, as well as the droplet-epitaxy-based techniques [5-10], both based on molecular beam epitaxy (MBE) fabrication. In comparison to Stranski-Krastanov growth, droplet epitaxy is more flexible regarding the choice of materials. Moreover, the fabrication of unstrained nanostructures is possible.

A central point for droplet epitaxy is the agglomeration of the planarly deposited material into spatially well-separated droplets. The driving force for droplet formation in the Volmer-Weber growth mode [11] is the minimization of the surface energy of the deposited droplet material. In this sense, droplet-based techniques require as the central prerequisite a dewetting character of the solid-liquid interface.

**Figure 1:**
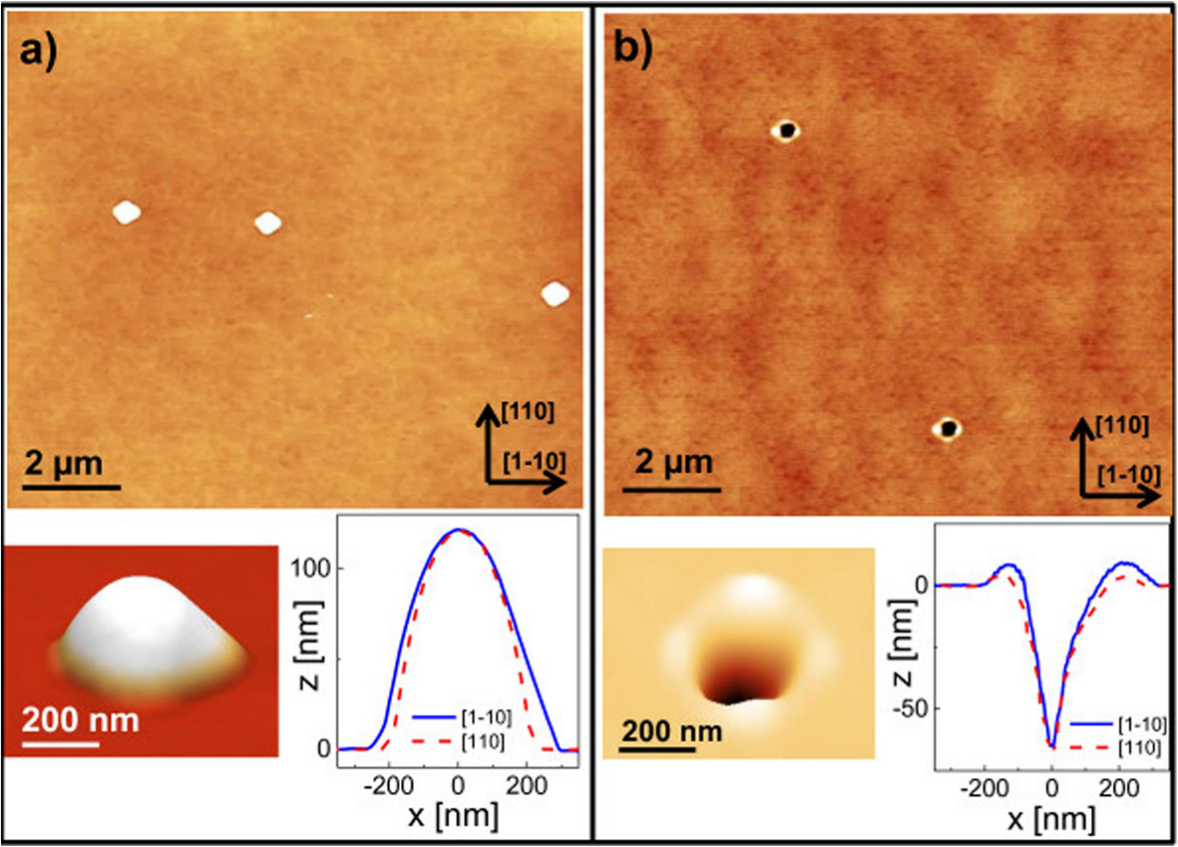
**Example for the transformation of as-grown droplets into nanoholes with walls during post-growth annealing. (a)** AFM micrograph of a GaAs surface with droplets after deposition of 2.0 ML of Ga at *T*=650°C without annealing together with a perspective view and linescans of a single droplet. **(b)** GaAs surface with nanoholes after Ga droplet deposition and 120-s annealing.

After deposition, the material localized in the droplets is functionalized for nanostructure creation. In the most widely used approach, group III metal droplets are crystallized under a group V atmosphere to form III/V semiconductor quantum dots [6-10], quantum dot molecules [12], or quantum ring complexes [13-17]. An alternative approach using a low group V flux is the local droplet etching (LDE), where group III metal droplets drill nanoholes into III/V-semiconductor surfaces [18-25]. An example for a surface with nanoholes after droplet etching is shown in Figure 1b.

Functionalized nanostructures arise when the holes are refilled with material in a subsequent epitaxy step. From the perspective of applications, local droplet etching introduces a novel degree of freedom for the self-assembled patterning of semiconductor surfaces using conventional molecular beam epitaxy technology. The process works with a number of different materials, such as Ga, Al, In, InGa, and AlGa droplets on GaAs, AlGaAs, and AlAs substrates [18,21,24,26]. By the filling of droplet-etched nanoholes with a material different from the substrate, the fabrication of novel types of nanostructures has been demonstrated, such as localized InAs quantum dots [22], strain-free GaAs hole quantum dots [27-30], vertically stacked quantum dot pairs [31], and ultra-short nanopillars for thermal and electron transport experiments [32-34].

Regarding the fabrication processes, the central parameters of droplet etching which differ from droplet epitaxy involve a low group V flux to avoid crystallization of the droplets, as well as higher temperatures allowing substantial substrate etching and material removal. As a result, during droplet epitaxy, the initial droplet shape is mostly conserved, whereas during droplet etching, the droplets are mostly removed together with an amount of substrate material. The present paper discusses experimental results on the dynamics during the surface mass transportation. Moreover, it introduces a simple model that illuminates the basic etching mechanisms and allows estimation of the process-parameter-dependent hole depth.

## Methods

**Figure 2:**
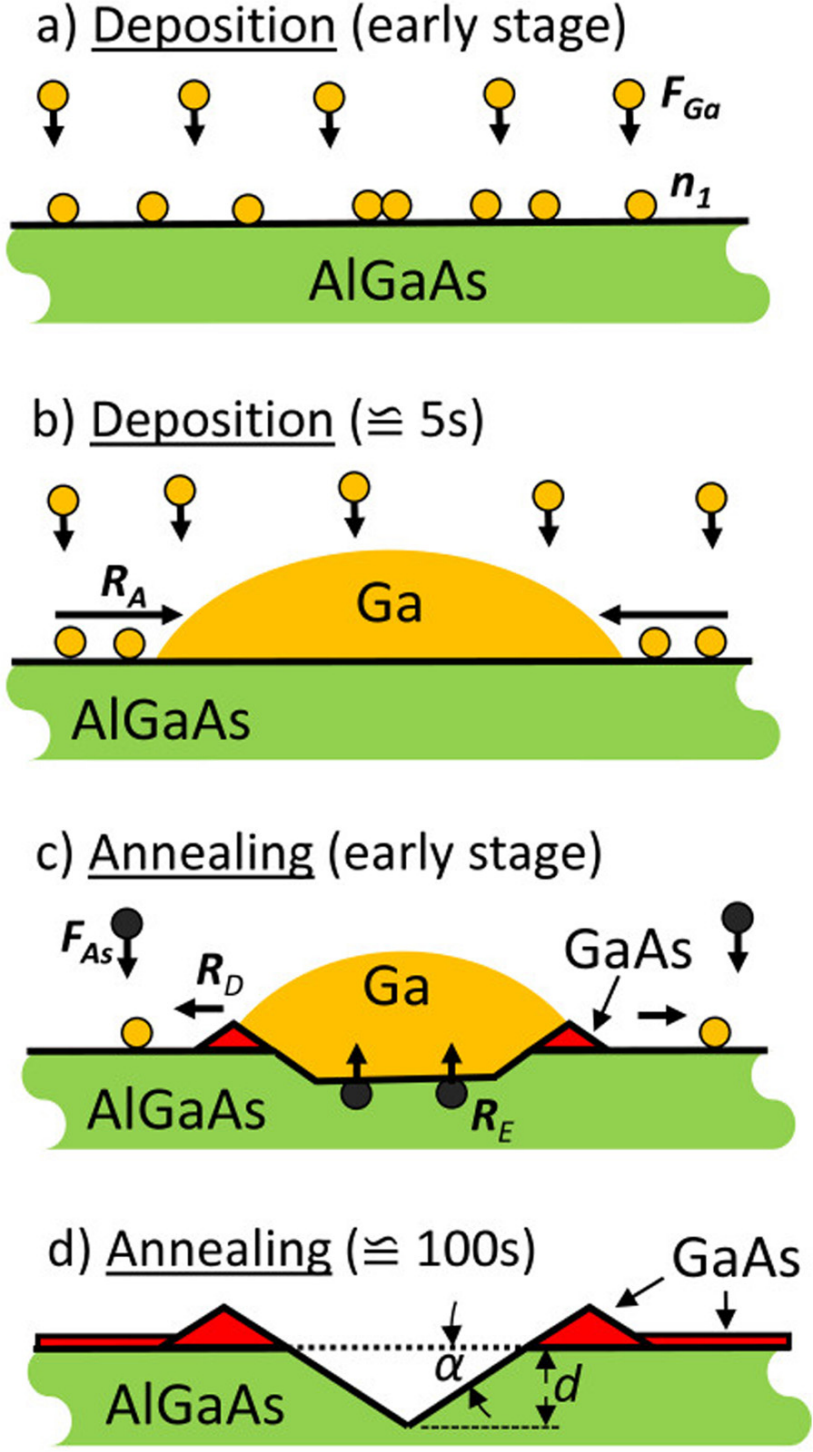
**Schematic representation of the different steps of a Ga on AlGaAs droplet etching process. (a)** Planar deposition of Ga with flux *F*_Ga_ yielding an increase of the Ga adatom density *n*_1_. Ga droplets are nucleated by collisions between diffusing Ga adatoms. **(b)** Droplet shape establishment with increasing coverage and increase of the droplet volume by adatom attachment with rate *R*_*A*_. **(c)** Etching and removal of substrate material by As diffusion with rate *R*_*E*_ and droplet material detachment with rate *R*_*D*_ during post-growth annealing. The detached Ga atoms crystallize a thin GaAs layer with background As of flux *F*_As_. **(d)** Final hole with depth *d* and side-facet angle *α* surrounded by a GaAs wall.

The samples are fabricated using a solid-source MBE system equipped with a valved cracker source for As _4_. A droplet-etching process takes place in two steps. First, droplet material is deposited uniformly over the substrate and droplets are formed in the Volmer-Weber growth mode driven by a minimization of the surface energy (Figures 1a, 2a,b). Here, we used a growth rate of 0.8 monolayers per second (ML/s) for Ga and 0.4 ML/s for Al. The coverage *θ* = 1 − 2 ML with droplet material is adjusted by the deposition time. In a subsequent post-growth thermal-annealing step of 120 to 180 s, the droplets transform into nanoholes surrounded by walls (Figures 1b, 2c,d). In the present experiments, we chose equal temperatures *T* for droplet deposition and annealing. The As flux is reduced to about 1 × 10^−7^ Torr by closing the As cell shutter and valve. During annealing, in addition, the main shutter in front of the sample is closed. In some experiments, the As background flux is further reduced as will be described below.

The morphology of nanoholes formed by local droplet etching are characterized using atomic force microscopy in tapping mode under ambient atmosphere. For the materials discussed here, we see no influence of oxidation. In contrast to that, earlier droplet-etching studies on AlAs surfaces exhibit fast and strong oxidation of the holes under air [27].

## Results and discussion

In the present experiments, no indications for droplet motion were found. Spontaneous running of Ga droplets was observed on annealed GaAs surfaces in the regime of incongruent evaporation [35]. In comparison to the experiments on GaAs, the present AlGaAs surfaces are thermally more stable with a critical temperature for incongruent evaporation which is assumed to be above the temperatures used here.

The equilibrium shape of the droplets at the end of the deposition step (Figures 1a, 2b) can be characterized by the contact angle *Θ*_C_, which is related to the energies of the respective interfaces via Youngs’s equation *γ*_S_−*γ*_SL_=*γ*_L_ cos(*Θ*_C_), with the energy *γ*_S_ of the solid-vacuum interface, the energy *γ*_L_ of the liquid-vacuum interface, and the energy *γ*_SL_ of the solid-liquid interface. Measured values of the contact angle and of the Ga interface energies are given in Table 1.

**Table 1:** **Contact angles for Ga and Al droplets at the end of the deposition step**

	***Θ***_**C**_	***γ***_**L**_	***γ***_**S**_	***γ***_**SL**_
Drop. - Sub.	degree	[J/m ^2^]	[J/m ^2^]	[J/m ^2^]
Ga - (Al)GaAs	45…55	0.67 [36]	1.04 [37]	0.61
Al - AlGaAs	60…70	-	-	-

Contact angles *Θ*_*C*_ at *T*=600°C for Ga and Al droplets with *θ*=1…2 ML on AlGaAs and GaAs substrates at the end of the deposition step without annealing. The values of *Θ*_C_ are determined from AFM measurements of the droplet height and radius under the assumption that the droplets are shaped like segments of a sphere [21]. For Ga droplets with an average *θ*_C_ = 50°, the energy *γ*_SL_ of the solid-liquid interface is estimated from Youngs’s equation using literature values of *γ*_L_ and *γ*_S_.

The essential processes for droplet etching, i.e., the etching of the substrate and the removal of the material from the initial droplet position, take place during the post-growth annealing step. A central process for etching is that material from the crystalline substrate is removed by diffusion of As into the liquid droplet material driven by the concentration gradient (Figure 2c) [38]. As a consequence, the substrate becomes liquid at the interface to the droplet and the droplets quasi sink into the substrate.

As an important point, the solubility of As in the droplet material is limited to a maximum of about 10 ^−4^ [39]. As a consequence, etching would stop very fast without a mechanism removing As from the droplets. We identify the formation of the crystalline wall around the nanohole opening to be the essential process for As removal [38]. That means, after removal from the substrate, the As atoms travel very fast through the liquid droplet [40] and crystallize the wall [38,41] with droplet material at the triple line at the border between the droplet surface and the substrate. This picture is supported by the observation of equal volumes of material stored inside a wall and of material removed from a hole [42]. Since the wall is composed of Arsenides of the droplet material, etching with Ga droplets yields GaAs walls that act as quantum rings [21,26], whereas etching with Al droplets yields optically inactive AlAs walls [27]. A model describing the etching process and wall crystallization is described in [38].

Nevertheless, the wall crystallization removes only a few percent of the initial droplet material at the beginning of the annealing step [42]. Therefore, an additional process is necessary to uncover the etched holes below the droplets. While in a previous model with different focus [38] it has been suggested that droplet material might be removed by desorption, recent results indicate rather that the droplet material detaches from the initial droplet positions and uniformly spreads over the substrate [34]. These experiments will be discussed in the following. A similar behavior with adatom detachment and spreading into ring structures was also observed during droplet epitaxy at *T*<400°C [43].

**Figure 3:**
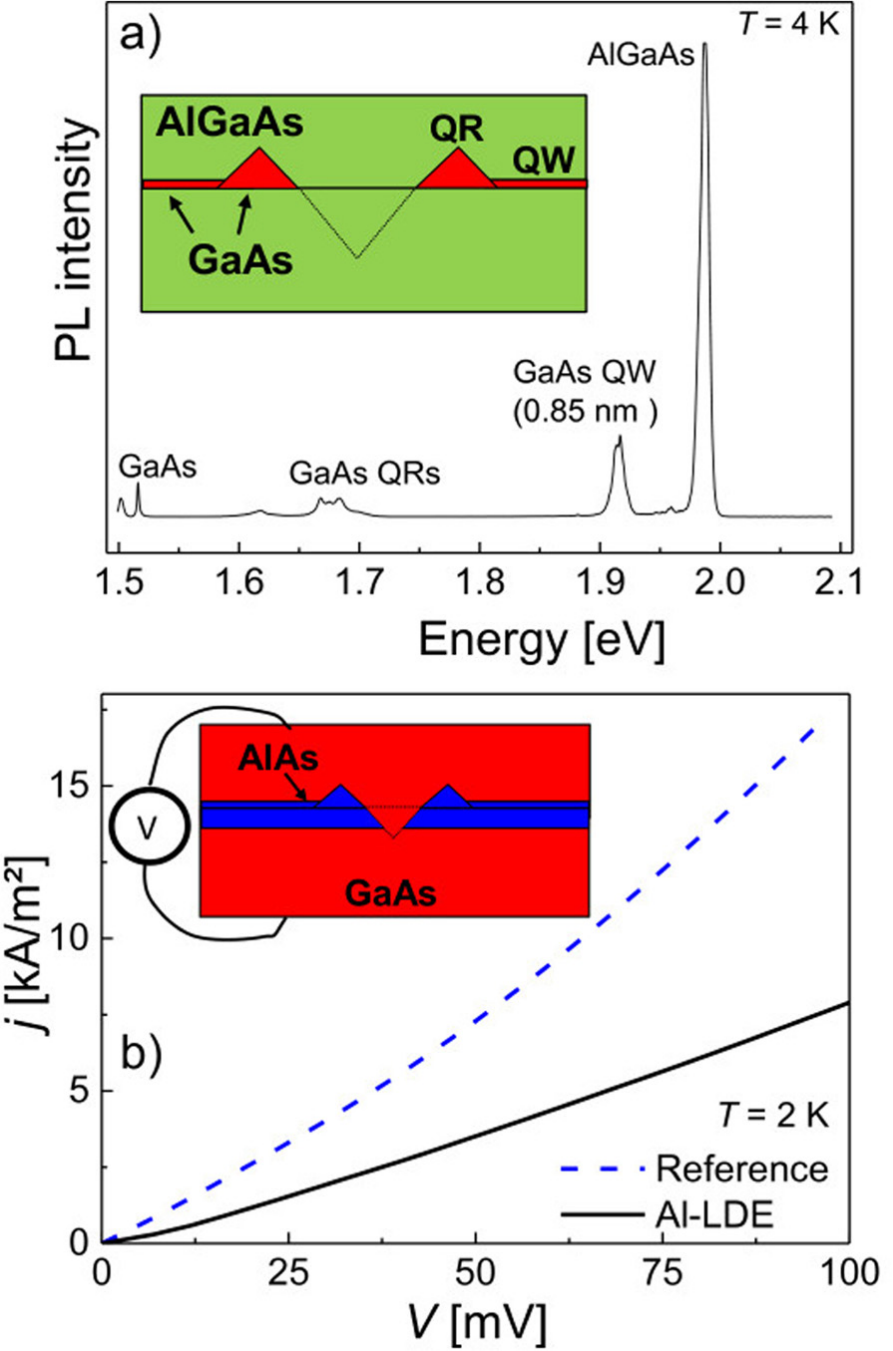
**Photoluminescence and transport experiments establishing the droplet material spreading over the surface. (a)** Photoluminescence (PL) measurement of a GaAs quantum ring (QR) sample fabricated using Ga LDE. The insert shows a schematic sample cross section. In addition to the weak QR signal, a peak at 1.917 V indicates the presence of a uniform GaAs quantum well (QW) with thickness of 0.85 nm. Importantly, the volume of the additional planar layer agrees with the amount of deposited droplet material. **(b)** Current density *j* over a 8-nm-thick AlAs tunnel barrier (reference) compared to a sample, where the tunnel barrier has been thickened by an additional Al-LDE step. The insert shows a schematic sample cross section.

Figure 3a shows an ensemble photoluminescence (PL) spectrum from a GaAs quantum ring (QR) sample with a schematic layer sequence shown in the inset. The rings are fabricated by Ga droplet etching of an AlGaAs substrate and subsequent overgrowth with AlGaAs [21]. As discussed above, etching with Ga droplets yields crystalline GaAs walls around the nanohole openings which can be regarded as quantum rings. In the PL data, the QR emission is visible at *E*= 1.67…1.68 eV. Additional micro-PL measurements (not shown here) indicate that the QR emission is spatially localized on the surface with feature density equivalent to the hole density. In addition to the localized QR-related signals, the PL spectrum shows a peak at 1.915 eV uniformly over the substrate surface that we attribute to a GaAs quantum well (QW). Importantly, the QW thickness is in agreement with the amount of deposited droplet material. This indicates mass conservation of the initial droplet material after spreading over the sample surface and formation of a uniform GaAs QW.

In a further experiment, we have measured the tunnel current density *j* over a 8-nm-thick AlAs barrier in a reference sample and over a 8-nm-thick AlAs barrier with additional Al-LDE step in a further sample. The inset in Figure 3b shows a scheme of the later sample. The LDE-holes in the second sample are filled with GaAs and act as quantum point-contacts. The current density data demonstrates that, surprisingly, the sample containing point-contact holes in the barrier has a higher resistance than the reference (Figure 3b). This result indicates that the current density *j* is dominated by electron tunneling through the barrier and that the tunnel barrier is thickened by the Al-LDE process [34]. We conclude that also Al as a droplet material detaches from the droplet during annealing and spreads uniformly over the substrate surface.

The above results establish the interesting mechanism that the planarly deposited material evolves over localized droplets finally back into a planar distribution (Figure 2).

**Figure 4:**
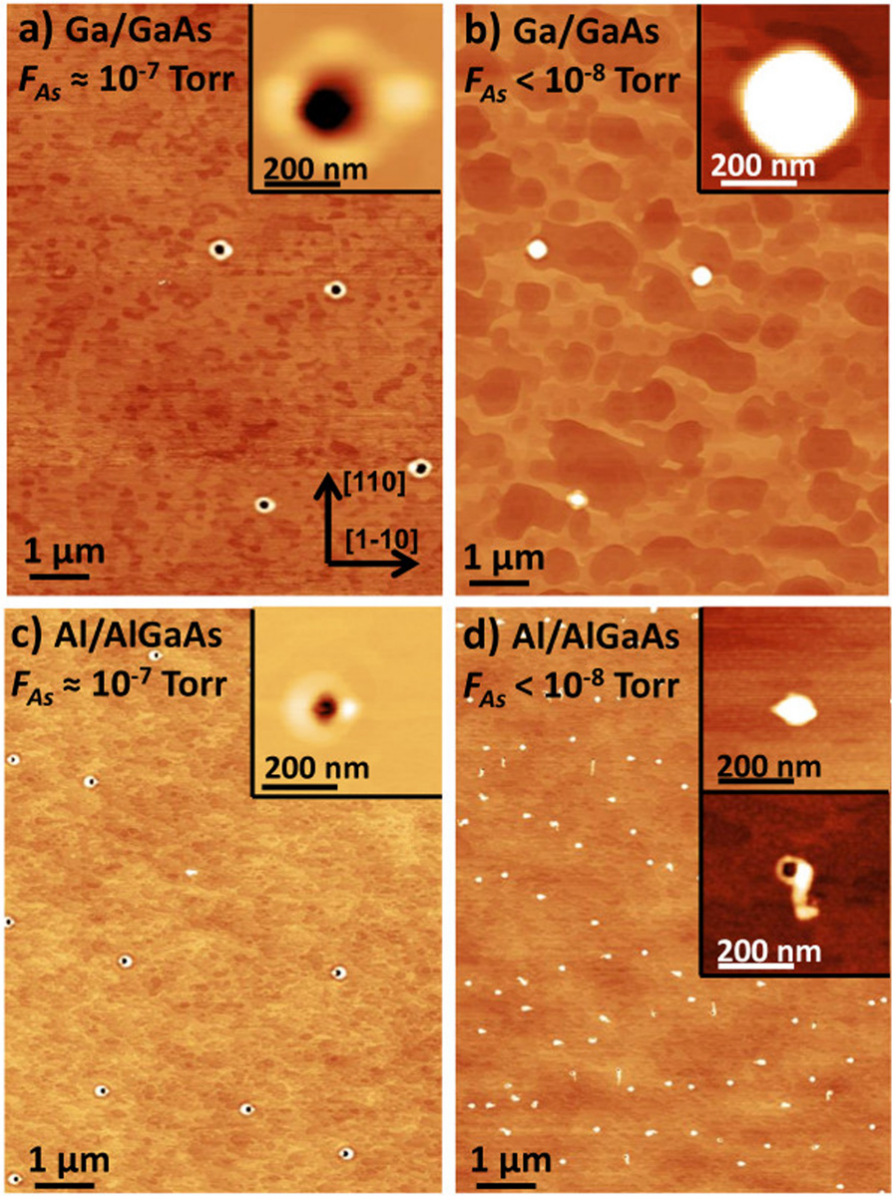
**AFM images demonstrating droplet conservation at completely minimized As background. (a)** GaAs surface after Ga-LDE with *θ* = 2.0 ML and *T*=600°C. The As flux *F*_As_ during annealing is of about 1 ×10^−7^ Torr according to our typical process conditions. **(b)** GaAs surface after Ga-LDE at minimized As background flux *F*_As_<1×10^−8^ Torr by a 1-h growth interruption with the As cell switched off before etching. **(c)** AlGaAs surface after Al-LDE with *θ* = 1.0 ML, *T*=640°C, and *F*_As_≃1×10^−7^ Torr. **(d)** AlGaAs surface after Al-LDE at *F*_As_<1×10^−8^ Torr.

In the next experiments, we have studied the influence of the background As flux *F*_As_ on nanohole formation. Typically, we use an As flux of about 10 ^−7^ Torr during droplet deposition and post-growth annealing. Corresponding surfaces with nanoholes formed by etching with Ga and Al droplets are shown in Figure 4a,c, respectively. Now, we have minimized *F*_As_ before etching to less than *F*_As_<1×10^−8^ Torr during a 1-h growth interruption with the As cell switched off. Importantly, here, the surfaces are covered with droplets, and nanohole formation is nearly suppressed (Figure 4b,d). Small holes are visible only on the AlGaAs surface that we attribute to defects caused by contamination of the highly reactive AlGaAs surface during the 1-h growth interruption. These results are in agreement with a very recent study of Fuster et al. [44], which shows that droplet etching with Ga at *T*=500°C is only possible in the presence of an arsenic flux. Additional experiments [42] show that hole formation is also suppressed for *F*_As_>3×10^−6^ Torr and that flat surfaces are formed, instead. These results establish a range of $2\times 10^{-6} > F_{\text {As}} \gtrapprox 1\times 10^{-8}$ Torr suitable for droplet etching.

**Figure 5:**
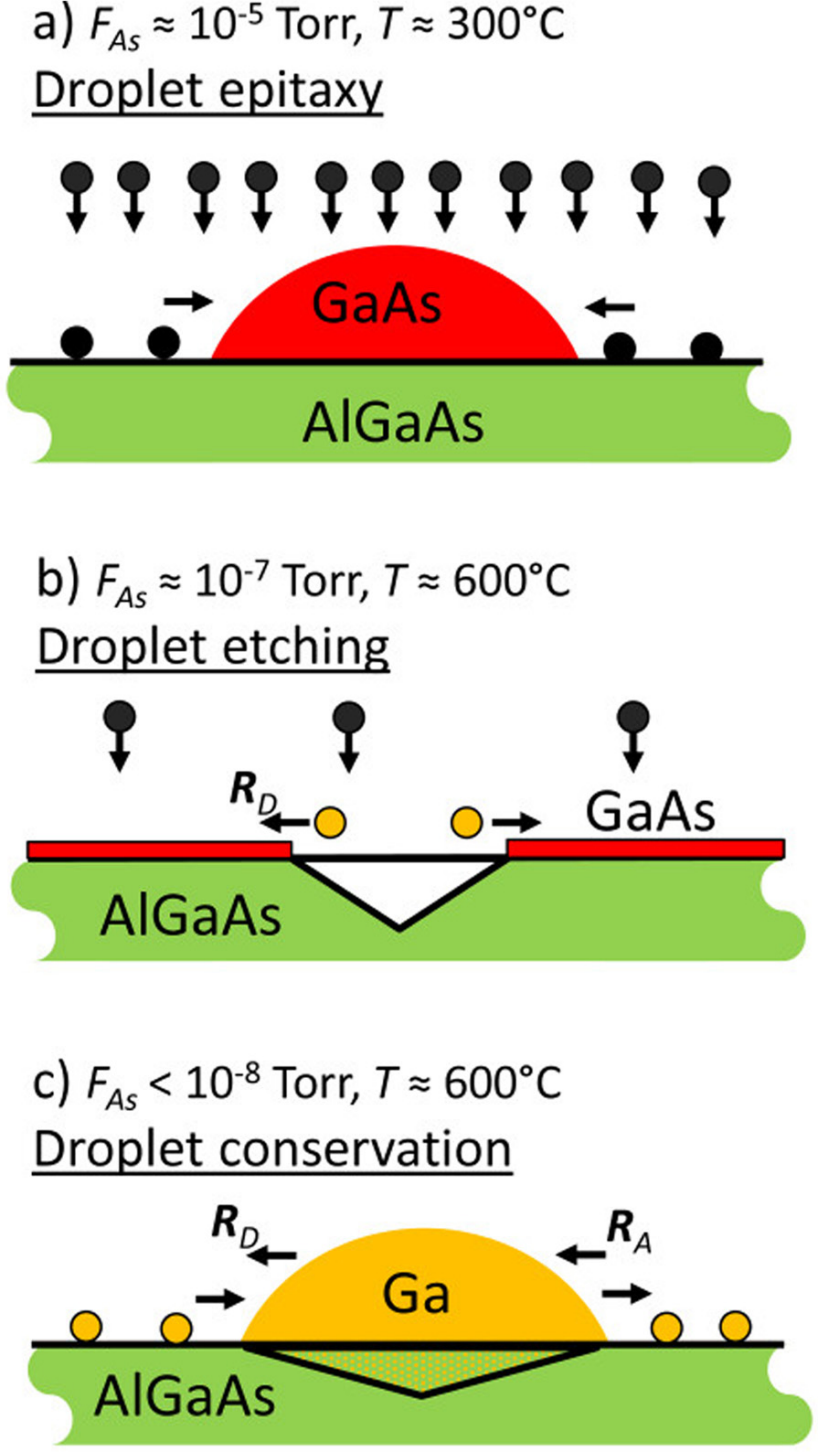
**Scheme of droplet-epitaxy-based regimes at different As fluxes** ***F***_**As**_ **and process temperatures** ***T*** **. (a)** Droplet epitaxy with crystallization of GaAs QDs using a high *F*_As_. **(b)** Droplet etching of nanoholes at high *T* and small *F*_As_. Here, Ga atoms detaching from the droplet crystallize with background As and form a thin GaAs layer. **(c)** Droplet conservation at high *T* and minimized *F*_As_. Adatom detachment from and re-attachment to the droplets is balanced.

Figure 5 summarizes different droplet-epitaxy-based regimes as a function of process temperature *T* and As flux *F*_As_. At *T*≃300°C and *F*_As_≃1×10^−5^ Torr, the high As flux crystallizes the droplets into semiconducting GaAs quantum dots or rings in the droplet epitaxy regime. As an interesting point, cross-sectional scanning tunneling microscopy (X-STM) experiments indicate substrate liquefaction below the droplets already at this low temperature [45]. At higher *T*≃600°C and lower *F*_As_≃1×10^−7^ Torr, nanoholes are formed by droplet etching. Here, the droplets are not crystallized, but instead, most of the droplet material diffuses away from the initial droplet position and spreads over the substrate. Substrate liquefaction and, thus, etching is significant due to the high temperature. And finally, at *T*≃600°C and minimized *F*_As_<1×10^−8^ Torr, the droplets are conserved. We assume here a balance between detachment of material from and re-attachment to the droplets, as will be discussed below in more detail. The substrate below the droplet is probably liquefied, but the hole is not uncovered due to the missing droplet material removal.

We introduce now a simple model of the droplet-etching process which assumes that the droplets are already nucleated and describes the dynamics during progressed droplet deposition (Figure 2b) and during annealing (Figure 2c). A model of droplet nucleation (Figure 2a) has been discussed previously [8]. As a starting point, we assume an array of droplets of identical size. The droplets are characterized by their density *N* in units of droplets per lattice site and dimensionless average volume *V*≃*θ*/*N* in units of the number of atoms inside a single droplet, with the droplet material coverage *θ* in monolayers (ML). As a simplification under negligence of the Ostwald ripening [46], we assume a constant droplet density [47]. In addition to the droplets, Ga adatoms with density *n*_1_ per lattice site are located on the surface.

The average droplet volume *V* is modified by attachment and detachment of atoms. In particular, attachment of mobile adatoms to a single droplet takes place with a thermally activated rate *n*_1_*V*^1/3^*R*_*A*_ (Figure 2b), where *R*_*A*_=*ν* exp[−*E*_*A*_/(*k*_*B*_*T*)], *ν* is a vibrational frequency, *E*_*A*_ is the activation energy for attachment, *k*_*B*_ is Boltzmann’s constant, and *T* the temperature. In addition, detachment of atoms located at the contact line between the droplet surface and the substrate surface is possible with rate *V*^1/3^*R*_*D*_, where *R*_*D*_=*ν* exp[−*E*_*D*_/(*k*_*B*_*T*)], and *E*_*D*_ is the activation energy for detachment. The resulting droplet volume evolution is described by:

(1) $$ \frac{d V} {dt} = n_{1} V^{1/3} R_{A} - V^{1/3} R_{D}  $$

The monomer density is balanced by the impinging Ga flux *F* and the above attachment and detachment processes, as well as by re-evaporation with rate *R*_*R*_ and reaction with background As flux *F*_As_:

(2) $$ \frac{d n_{1}} {dt} = F_{\text{Ga}} + N V^{1/3} (R_{D} - n_{1} R_{A}) - n_{1} R_{R} - n_{1} \sigma_{\text{As}} F_{\text{As}}  $$

where *F*_Ga_ is the Ga flux and *σ*_As_ represents a reaction cross section. Considering the mass conservation indicated by the experiments shown in Figure 3, we assume that re-evaporation is negligibly small so that *n*_1_*R*_*R*_≃0.

We will now discuss several regimes of the incident beam fluxes based on Equations 1 and 2:

1. *F*_Ga_<*F*_As_: this is the usual GaAs growth regime with As overpressure. Here, neither droplets nor nanoholes are formed.
2. *F*_Ga_>*F*_As_: this is a growth regime used for the generation of Ga droplets. The excess Ga first increases the surface adatom density *n*_1_ according to Equation 2 at *N*≃0, and droplets are nucleated by collisions between diffusing adatoms (Figure 2a). Later, the droplet volume increases due to the attachment of mobile adatoms according to Equation 1 (Figure 2b). Detachment of atoms from the droplets is negligible at this stage.
  In the transition regime between regimes 1 and 2 at a relatively high *F*_As_, the value of *dV*/*dt* might become very small since most of the deposited Ga is directly incorporated into the substrate without forming droplets large enough for etching [42]. This sets the upper limit in the As flux for the observation of droplet etching phenomena.
3. *F*_Ga_=0,*F*_As_≫0: this is an annealing regime under high As background flux as used for droplet crystallization in droplet epitaxy (Figure 5a). A model describing the mechanisms of droplet epitaxy in this regime is given in [41].
4. *F*_Ga_=0,*F*_As_>0: this is an annealing regime under small As background flux as used for droplet etching (Figure 5b). Ga atoms detached from the droplets react with arsenic with rate *n*_1_*σ*_As_*F*_As_>0 and form a planar GaAs layer (Figure 3a). As consequences, *n*_1_≃0, re-attachment of adatoms becomes negligibly small *n*_1_*V*^1/3^*R*_*A*_≃0, and thus, the droplets shrink *dV*/*dt*<0. This droplet material removal is essential to uncover the etched nanoholes.
5. *F*_Ga_=0,*F*_As_=0: this is an annealing regime under completely minimized As background flux (Figure 5c). Here, the balance *n*_1_*R*_*A*_=*R*_*D*_ between attachment and detachment of atoms conserves the droplet volume *dV*/*dt* = 0.

A simple model of the nanohole depth is proposed considering droplet etching regime 4. As described above, a small *F*_As_ is applied, yielding negligible adatom re-attachment. This simplifies Equation 1 to *dV*(*t*)/*dt*=−*V*^1/3^*R*_*D*_. Furthermore, a droplet lifetime *t*_*R*_ up to complete removal of the droplet material *V*(*t*_*R*_)=0 is introduced. This yields *t*_*R*_∝*V*(0)^2/3^/*R*_*D*_, with the droplet volume *V*(0)=*θ*/*N* at the end of the deposition stage, the deposited droplet material coverage *θ*, and the droplet density *N*. We assume now that the average depth *d*=*R*_*E*_*t*_*R*_ of the nanoholes is given by the etching rate *R*_*E*_=*ν* exp[−*E*_*E*_/(*k*_*B*_*T*)] and the etching time *t*_*R*_, where *E*_*E*_ is the etching activation energy characterizing a thermally activated emission of As atoms from the crystalline substrate into the liquid droplet (Figure 2c). Furthermore, we consider classical nucleation theory [48,49] for the density of the droplets *N*∝ exp[−*E*_*N*_/(*k*_*B*_*T*)], with the nucleation-related characteristic energy *E*_*N*_. Previous experiments have established that *N* does not depend on *θ* [47]. This approach yields for the hole depth:

(3) $$ d = c_{h} \theta^{2/3} \exp [- E_{h}/(k_{B} T)]  $$

with constant *c*_*h*_ and *E*_*h*_=*E*_*E*_−*E*_*D*_+2*E*_*N*_/3. This simple scaling model allows the analytical calculation of the depth of droplet-etched nanoholes as a function of the most relevant process parameters temperature and droplet material coverage. More detailed numerical models which consider in addition the hole morphology including the wall are discussed in the complementary references [38,50], both relying on similar assumptions. In [38], the temperature-dependent hole morphology is modelled, whereas [50] models the influence of *F*_As_ but without considering the temperature dependence.

**Figure 6:**
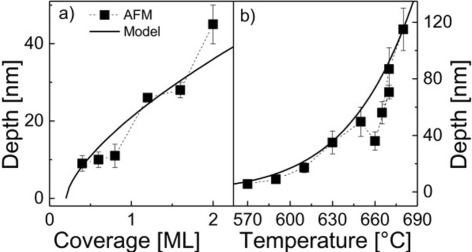
**Measured and calculated depth** ***d*** **of Al droplet etched nanoholes. (a)** Comparison of measured and calculated depth *d* of Al-droplet-etched nanoholes as a function of the Al coverage *θ*. The model results (line) are calculated using Equation 3, and the experimental data (symbols) are taken from [47]. **(b)** Measured and calculated depth *d* of Al-droplet-etched nanoholes as a function of the process temperature *T*.

Figure 6 shows a comparison of measured and calculated values of *d* for LDE with Al droplets on AlGaAs as a function of *T* and *θ*. For the calculations, we use *c*_*h*_ =1.1 ×10^11^ nm ML ^−2/3^ and *E*_*h*_ = 1.73 eV. The coverage is given by *θ*=*θ*_0_−*θ*_*c*_ considering that an amount of *θ*_*c*_ = 0.2 ML is consumed by the surface for a reconstruction change [47], with the deposited amount of droplet material *θ*_0_=*Ft*. The good agreement supports the validity of the model and its possibility to predict the nanohole properties.

**Figure 7:**
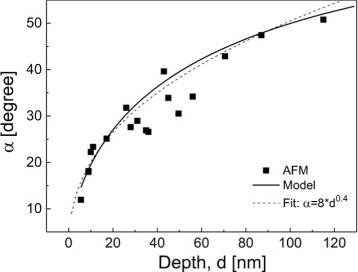
**Measured and calculated hole side-facet angle** ***α*** **as function of the hole depth.** Every data point represents the average over a sample with *d* varied by changing *T* and *θ*. In addition to calculations done using a model described in [47], results of a simple power-law fit are shown.

For a complete characterization of the nanoholes, assuming as approximation an inverted cone-like shape (Figure 2d), in addition to the depth also, either the radius *r*=*d*/ tan*α* of the hole opening or the angle *α* between the hole side-facet and the flat surface is required. Figure 7 shows measured values of *α* for nanoholes where *d* was varied by the process parameters *T* and *θ*. Interestingly, the data indicate a systematic increase of *θ* with increasing *d* which is well reproduced by an empirical power law *α*≃8*d*^0.4^. Furthermore, Figure 7 demonstrates that an extension of the above model of the hole depth described in [47] also agrees well with the *α* vs. *d* data.

## Conclusions

The mechanisms behind the self-assembled etching of nanoholes into semiconductor surfaces through liquid metal droplets are studied. As a central finding, we observe that a small arsenic background flux is essential for etching. This As flux crystallizes atoms detaching from the droplets in the form of a uniform GaAs or AlAs layer. Otherwise, using a completely minimized As flux, the detached atoms will re-attach to the droplet and conserve it. On the other hand, an As flux being too high will also suppress nanohole etching [42]. These results indicate a complex interplay between crystallization processes as well as adatom detachment from and re-attachment to the droplets and suggests the As pressure as an additional important process parameter for nanohole tuning.

A simple model is proposed that explains the mechanisms behind the surface mass transport during local droplet etching. Furthermore, the model allows an easy prediction of the nanohole structural properties, and in particular, a quantitative reproduction of experimental values of the nanohole depth as a function of the process parameters is demonstrated.
